# Sub-Convulsing Dose Administration of Pilocarpine Reduces Glycemia, Increases Anxiety-Like Behavior and Decelerates Cortical Spreading Depression in Rats Suckled on Various Litter Sizes

**DOI:** 10.3389/fnins.2018.00897

**Published:** 2018-12-03

**Authors:** Elian da Silva Francisco, Rubem Carlos Araújo Guedes

**Affiliations:** Departamento de Nutrição, Universidade Federal de Pernambuco, Recife, Brazil

**Keywords:** anxiety-like behavior, blood glucose, brain excitability, litter size, nutritional deficiency, pilocarpine, spreading depression, unfavorable lactation

## Abstract

Epilepsy and malnutrition constitute two worldwide health problems affecting behavior and brain function. The cholinergic agonist pilocarpine (300–380 mg/kg; single administration) reproduces the human type of temporal lobe epilepsy in rats. Pilocarpine-induced epilepsy in rodents has been associated with glycemia, learning and memory and anxiety disturbances. Cortical spreading depression (CSD) is a neural response that has been linked to brain excitability disorders and its diseases, and has been shown to be antagonized by acute pilocarpine. This study aimed to further investigate the effect of chronic pilocarpine at a sub-convulsing dose on weight gain, blood glucose levels, anxiety-like behavior and CSD. In addition, we tested whether unfavorable lactation-induced malnutrition could modulate the pilocarpine effects. Wistar rats were suckled under normal size and large size litters (litters with 9 and 15 pups; groups L_9_ and L_15_, respectively). From postnatal days (PND) 35–55, these young animals received a daily intraperitoneal injection of pilocarpine (45 mg/kg/day), or vehicle (saline), or no treatment (naïve). On PND58, the animals were behaviorally tested in an open field apparatus. This was immediately followed by 6 h fasting and blood glucose measurement. At PND60–65, CSD was recorded, and its parameters (velocity of propagation, amplitude, and duration) were calculated. Compared to the control groups, pilocarpine-treated animals presented with reduced weight gain and lower glycemia, increased anxiety-like behavior and decelerated CSD propagation. CSD velocity was higher (*p* < 0.001) in the L_15_ groups in comparison to the corresponding groups in the L_9_ condition. The results demonstrate an influence of chronic (21-day) administration of a sub-convulsing, very low dose (45 mg/kg) of pilocarpine on CSD propagation, anxiety-like behavior, glycemia and body weight. Furthermore, data reinforce the hypothesis of a relationship between CSD and brain excitability. The lactation condition seems to differentially modulate these effects.

## Introduction

Epilepsy is the third most common chronic brain disorder (Landgrave-Gómez et al., 2016), being more prevalent in developing countries (Hackett and Iype, 2001). The rodent model of epilepsy that is based on pilocarpine treatment has been widely used to reproduce the histological, biochemical, behavioral, and electrophysiological manifestations found in humans with temporal lobe epilepsy (Duarte et al., 2013). Pilocarpine is an alkaloid extracted from the leaves of the jaborandi plant (*Pilocarpus jaborandi*). It is a muscarinic non-selective cholinergic agonist capable of inducing status epilepticus in rodents with only a single intraperitoneal (i.p.,) dose (300–380 mg/kg) (Turski et al., 1989; dos Santos et al., 2000). Evidence suggests that epilepsy influences emotional responses in human beings (Devinsky, 2004; Jackson and Turkington, 2005), and anxiety has been the most common psychological disorder among people with epilepsy (Beyenburg et al., 2005). Pilocarpine-induced experimental epilepsy in rodents has been associated with glycemia, learning and memory disturbances and behavioral disorders such as depression and anxiety (Cardoso et al., 2009; Duarte et al., 2014; Castelhano et al., 2015; Cossa et al., 2016). Furthermore, although sub-convulsing doses of pilocarpine do not cause behavioral or electrocorticographic changes indicative of seizure, they are able to antagonize the propagation of the excitability-related phenomenon known as cortical spreading depression (CSD) along the cortical rodent tissue (Guedes and Vasconcelos, 2008).

First described by Aristides Leão, CSD represents a reduction in the spontaneous and evoked electrical activity of the cerebral cortex in response to an electrical, chemical, or mechanical stimulation of a point on the brain (Leão, 1944). This fully reversible neural response propagates very slowly (propagation velocity in the order of few mm/min) from the initially stimulated point to more and more remote parts of the tissue (Lima et al., 2017). CSD has been linked to brain excitability disorders and its diseases such as migraine with aura (Peeters et al., 2007; Vinogradova, 2018), multiple sclerosis (Pusic et al., 2015), epilepsy (Tamaki et al., 2017), traumatic brain injury (Mayevsky et al., 1998; Hartings et al., 2009), subarachnoid hemorrhage (Dreier et al., 2006; Sugimoto et al., 2016), and malignant ischemic stroke (Woitzik et al., 2013; Pinczolits et al., 2017). CSD has been demonstrated not only in experimental animals (Guedes et al., 2017; Accioly and Guedes, 2017) but also in humans (Carlson et al., 2016; Lauritzen and Strong, 2016). CSD propagation can be facilitated under unfavorable suckling conditions (pups being suckled in large litters; Lima et al., 2017), and this can modulate the effect of other treatments (Francisco and Guedes, 2015). Measuring CSD velocity of propagation along the cortical tissue has been largely used in our laboratory as a useful physiological index that helps to understand the electrophysiological aspects of brain functioning in health and disease (Guedes, 2011; Guedes et al., 2012). Increased or reduced CSD propagation velocity indicates a greater or lesser respective ability of the cortical tissue to propagate CSD, which can be associated with anxiety-like behavior (Francisco and Guedes, 2015; Lima et al., 2017) and glycemic changes (Francisco and Guedes, 2015; Souza et al., 2015).

In addition to epilepsy, malnutrition is an important public health problem in a number of developing countries, with economic and sociocultural implications. It is believed that the changes caused by both epilepsy and malnutrition, when in association, can potentiate their deleterious neural effects (Porto et al., 2010). However, the hypothesis that malnourished humans would present a higher incidence of epilepsy compared to well-nourished humans needs much investigation. Previous studies on well-nourished rats described significant effects of a convulsing dose of pilocarpine on body weight and glycemia (Cossa et al., 2016), as well as on the propagation of CSD (Guedes and Cavalheiro, 1997). However, it is not known whether this convulsing compound would act relevantly on behavioral and electrophysiological parameters, when chronically applied in very low sub-convulsing doses, i.e., when producing a situation of chronic muscarinic cholinergic activation. In this study, we investigated in the rat the repercussion of chronic administration of a sub-convulsing dose of pilocarpine on the functioning of neural tissue in order to answer the two following questions: (1) Does the chronic treatment with a sub-convulsing dose of pilocarpine induce glycemia and anxiety-like and electrophysiological (CSD) alterations? (2) Are such effects of pilocarpine influenced by unfavorable lactation conditions?

## Materials and Methods

### Animals

All experimental procedures were previously approved by the Institutional Ethics Committee for Animal Research of our University (Approval protocol no. 23076.015655/2015-99), whose norms comply with those norms established by the National Institutes of Health Guide for Care and Use of Laboratory Animals (Bethesda, MD, United States). Newborn Wistar rats of both genders, born from different dams, were assigned to be suckled under normal or unfavorable conditions, represented, respectively, by litters with nine pups (L_9_ groups) and litters with 15 pups (L_15_ groups). Weaning occurred on postnatal day (PND) 21, when pups were separated by sex and housed in polypropylene cages (51 cm × 35.5 cm × 18.5 cm; three rats per cage) under a 12-h light:12-h dark cycle (lights on at 6:00 a.m.), controlled temperature (23 ± 1°C), and with free access to water and the same commercial lab chow, with 23% protein, that was offered to their dams (Purina Ltd). In this study, we analyzed data from male pups only: 27 L_9_ and 27 L_15_ rats. The animals were weighed on PND7, PND21, PND35, PND49, and PND60.

### Administration of Pilocarpine

Pilocarpine hydrochloride and scopolamine methyl nitrate were purchased from Sigma-Aldrich (St Louis, MO, United States). From PND35 to PND55, L_9_ and L_15_ rats received a single daily intraperitoneal injection of pilocarpine (45 mg/kg/day dissolved in saline; *n* = 9 L_9_ and 9 L_15_ rats), as previously described (Guedes and Vasconcelos, 2008), or vehicle (saline; *n* = 9 L_9_ and 9 L_15_ rats). One additional L_9_ and one L_15_ group received no treatment (naïve groups; *n* = 9 L_9_ and 9 L_15_ rats). Scopolamine methyl nitrate, a muscarinic receptor antagonist, was administered i.p., (1 mg/kg/day dissolved in 0.9% saline) in both groups 30 min before pilocarpine or saline administration to prevent the peripheral cholinergic effects elicited by pilocarpine (Peixinho-Pena et al., 2012). Immediately following pilocarpine administration, the animals were observed over 1 h for detection of spontaneous seizures as measured by the Racine (1972) scale with the following stages: (0) No abnormality; (1) Mouth and facial movements; (2) Head nodding; (3) Forelimb clonus; (4) Rearing with forelimb clonus; (5) Rearing and falling with forelimb clonus. At this low dose of pilocarpine, no behavioral signs of epilepsy were detected in our animals.

### Open Field Test

On PND58, the rats were individually placed in the center of a circular arena (diameter 89 cm and height 52 cm). The apparatus was located in a room with dim light and sound attenuation. Rats were positioned in the center of the arena, and their movements were recorded using a digital camera for 5 min. Between each test, the open field apparatus was wiped with a paper cloth soaked with 70:30 ethanol:water solution. The video-recorded activity was stored in a computer and subsequently analyzed with the software ANY maze^TM^ (version 4.99 m), as previously described (Lima et al., 2017). The following parameters were considered: number of expelled fecal boluses, total distance traveled, total immobility time, number of entries in the central zone and the time spent in the central zone.

### Analysis of Blood Glucose

As previously reported (Francisco and Guedes, 2015), after the open field behavioral test, the animals were fasted for 6 h, after which a drop of blood was collected from the animal’s tail and used for measuring the blood glucose level using a portable glucose meter (G-TECH free).

### CSD Recording

On the day of the electrophysiological recording (PND60–PND65), each animal was anesthetized with a mixture of 1 g/kg urethane plus 40 mg/kg chloralose injected intraperitoneally. Three trephine holes were drilled on the right side of the skull, aligned in the frontal-to-occipital direction and parallel to the midline. One hole was positioned on the frontal bone (2 mm in diameter) and used to apply the stimulus (KCl) to elicit CSD. The other two holes were positioned on the parietal bone (3–4 mm in diameter) and used to record the propagating CSD wave. CSD was elicited at 30-min intervals by a 1-min application of a cotton ball (1–2 mm in diameter) soaked with 2% KCl solution (approximately 270 mM) to the anterior hole drilled at the frontal region. Rectal temperature was continuously monitored and maintained at 37 ± 1°C by means of a heating blanket. The DC slow potential change accompanying CSD was recorded for 4 h using two Ag–AgCl agar–Ringer electrodes (one in each hole) against a common reference electrode of the same type, placed on the nasal bones. We calculated the CSD velocity of propagation from the time required for a CSD wave to pass the distance between the two cortical electrodes. In the two recording locations, we used the initial point of each DC-negative rising phase as the reference point to calculate the CSD velocities. In addition, we calculated amplitude and duration of the CSD waves, as previously reported (Lima et al., 2017).

### Statistics

Results in all groups are expressed as the means ± standard deviations (SD). Body weight, anxiogenic-like behavioral activity, blood glucose level and CSD propagation rate were compared between groups using ANOVA, including the following as factors: lactation conditions (L_9_ and L_15_) and treatment (naïve, vehicle, pilocarpine), followed by a *post hoc* test (Holm–Sidak) where indicated. A *p* < 0.05 was considered significant.

## Results

### Body Weight

**FIGURE 1:**
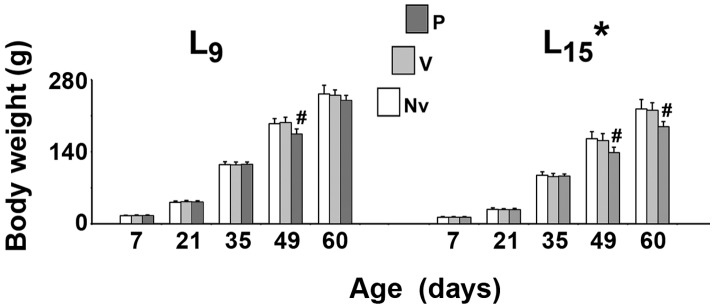
Suckling under large litter size and pilocarpine administration reduced body weight of male rats. Animals were previously suckled in litters with 9 and 15 pups (respectively, L_9_ and L_15_ condition). Naïve (Nv), no treatment; Vehicle (V), scopolamine methyl nitrate 1 mg/kg/day dissolved in 0.9% saline + 0.9% saline; Pilocarpine (P), scopolamine methyl nitrate 1 mg/kg/day + 45 mg/kg/day of pilocarpine; both dissolved in 0.9% saline. Note that since pilocarpine or vehicle intraperitoneal administration occurred from postnatal day 35–55, a differentiation between the three groups at day 7 and 21 can be done only by the assignment to the groups, but not by treatment. Data are mean ± standard deviation. ^∗^*p* < 0.001 compared with the corresponding L_9_ condition. #*p* < 0.001 compared with control groups in the same lactation condition (ANOVA plus Holm-Sidak test).

As shown in Figure 1, in all treatment groups ANOVA showed a main effect of the lactation condition on body weight (*p* < 0.001). The L_15_ animals presented with lower body weights compared with the L_9_ groups. The weight reduction ranged from 20.1 to 36.5% and was independent of the treatment. In the normal (L_9_) lactation condition, intergroup difference was observed in PND49 only [*F*(2,41) = 22.502; *p* < 0.001]. The treatment with pilocarpine was associated with weight reduction, compared to the respective L_9_ control groups. In the unfavorable (L_15_) lactation condition, pilocarpine reduced body weight at PND49 and PND60 [*F*(2,39) = 14.785; *p* < 0.001].

### Blood Glucose Level

**FIGURE 2:**
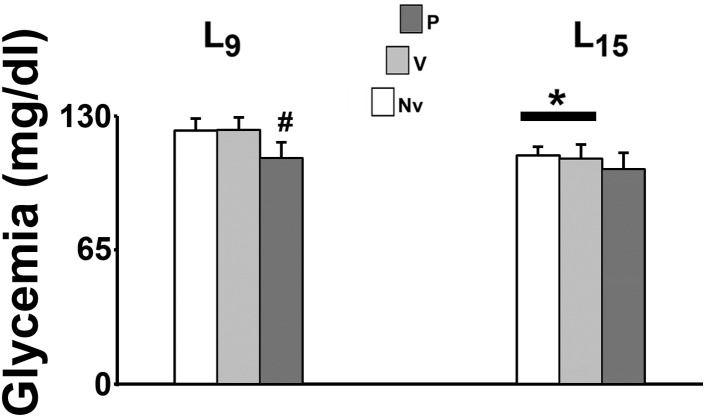
Suckling under large litter size and pilocarpine administration decreased blood glucose levels of 58 day-old male rats. Animals were previously suckled in litters with 9 and 15 pups (respectively, L_9_ and L_15_ condition). Naïve (Nv), no treatment; Vehicle (V), scopolamine methyl nitrate 1 mg/kg/day dissolved in 0.9% saline + 0.9% saline; Pilocarpine (P), scopolamine methyl nitrate 1 mg/kg/day + pilocarpine 45 mg/kg/day; both dissolved in 0.9% saline. Pilocarpine or vehicle intraperitoneal administration occurred from postnatal day 35–55. Data are mean ± standard deviation. ^∗^*p* < 0.001 compared with the corresponding L_9_ condition. #*p* < 0.001 compared with control groups in the same lactation condition (ANOVA plus Holm-Sidak test).

In the L_15_ control groups, glycemia was significantly lower than the corresponding L_9_ groups [*F*(1,35) = 22.990; *p* < 0.001]. Pilocarpine treatment reduced blood glucose levels in the L_9_, but not in the L_15_ groups [*F*(2,35) = 9.709; *p* < 0.001] compared with the corresponding control groups (naïve and vehicle). Data on glycemia are illustrated in Figure 2.

### Behavioral Activity in the Open Field

**FIGURE 3:**
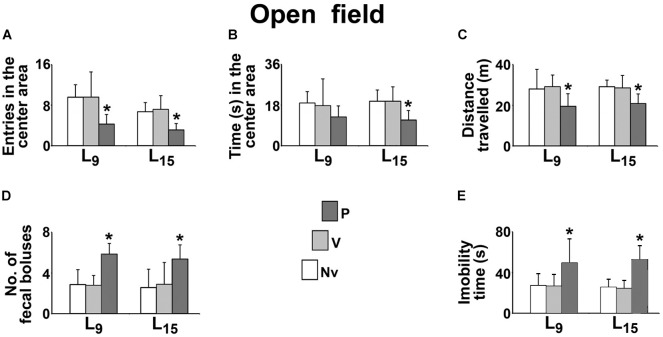
Sub-convulsing dose administration of pilocarpine results in anxiogenic-like behavioral activity in the open field test of 58 day-old male rats previously suckled in litters with 9 and 15 pups (respectively, L_9_ and L_15_ condition). Anxiogenic-like activity was characterized as a decrease of the following parameters: number of entries into the central area **(A)**, time spent in the central area **(B)** and distance traveled **(C)**, as well as an increase of the number of fecal boluses expelled **(D)**, and immobility time **(E)**. Naïve (Nv), no treatment; Vehicle (V), scopolamine methyl nitrate 1 mg/kg/day dissolved in 0.9% saline + 0.9% saline; Pilocarpine (P), scopolamine methyl nitrate 1 mg/kg/day + 45 mg/kg/day of pilocarpine; both dissolved in 0.9% saline. Pilocarpine or vehicle intraperitoneal administration occurred from postnatal day 35–55. Data are mean ± standard deviation. ^∗^*p* = 0.007 for time in the center area compared with control groups in the same lactation condition and *p* < 0.001 for other parameters (ANOVA plus Holm-Sidak test).

The effect of administration of pilocarpine on the behavioral activity in the open field test is shown in Figure 3. Compared with the naïve (Nv) and vehicle-treated (V) controls, the L_9_ group treated with pilocarpine (P) entered in the center area a lower number of times (P, 4.3 ± 1.9 vs. Nv, 9.6 ± 2.4 and V, 9.6 ± 5.0; *p* < 0.001), expelled a higher number of fecal boluses (P, 5.9 ± 1.1 vs. Nv, 2.9 ± 1.5 and V, 2.8 ± 1.0; *p* < 0.001), traveled a shorter distance in the circular arena (P, 19.5 ± 6.2 m vs. Nv, 28.1 ± 9.7 m and V, 29.1 ± 5.8 m; *p* < 0.001) and remained in immobility for a longer time (P, 49.1 ± 23.9 s vs. Nv, 27.2 ± 11.8 s and V, 26.4 ± 11.7 s; *p* < 0.001). No difference was observed for the time in the center area (P, 12.7 ± 4.9 s vs. Nv, 18.9 ± 5.0 s and V, 17.7 ± 11.8 s).

In the L_15_ condition, the pilocarpine-treated group entered a lower number of times in the center area (P, 3.1 ± 1.3 vs. Nv, 6.7 ± 1.8 and V, 5.0 ± 2.7), remained a shorter time in the center area (P, 11.4 ± 4.1 s vs. Nv, 19.7 ± 4.9 s and V, 19.7 ± 6.4 s; *p* = 0.007), expelled a higher number of fecal boluses (P, 5.4 ± 1.4 vs. Nv, 2.6 ± 1.8 and V, 2.9 ± 2.2), traveled a shorter distance in circular arena (20.81 ± 4.70 m vs. 29.09 ± 3.31 m and 28.55 ± 6.04 m) and remained longer in immobility (P, 52.3 ± 13.9 s vs. Nv, 25.4 ± 8.2 s and V, 24.3 ± 8.2 s).

### CSD Parameters

**FIGURE 4:**
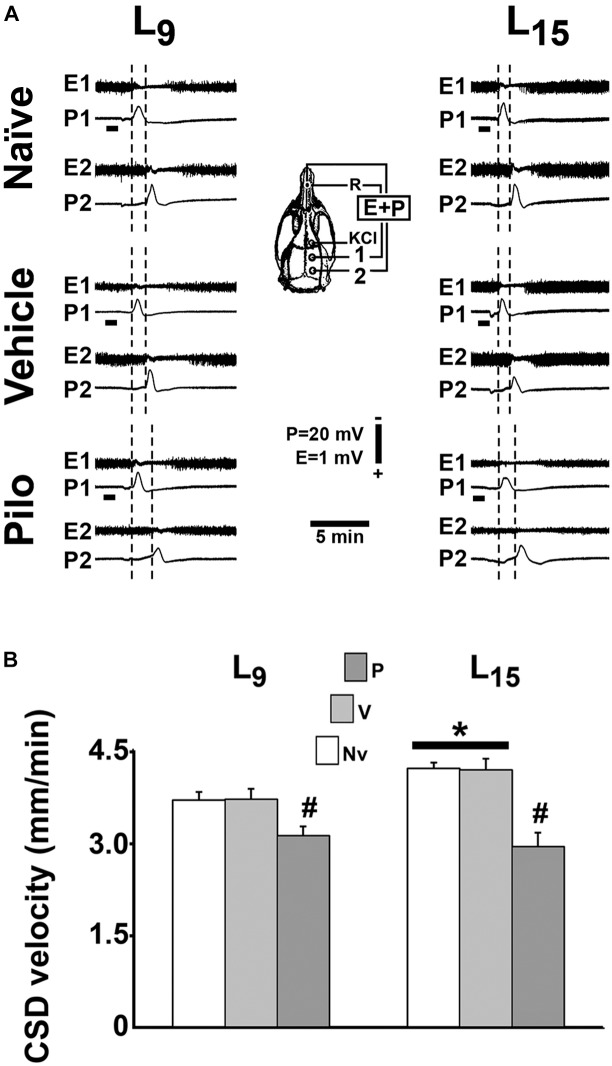
Repeated daily administration (21 days) of a sub-convulsing dose of pilocarpine reduced, whereas unfavorable lactation increased the propagation velocity of CSD in 60–65 day-old male rats previously suckled in litters with 9 and 15 pups (respectively, L_9_ and L_15_ condition). Pilocarpine or vehicle intraperitoneal administration occurred from postnatal day 35–55. **(A)** Electrocorticogram (E) and slow potential changes (P) during the passage of CSD, recorded at two cortical points (1 and 2) in three L_9_ and three L_15_ rats. The diagram of the skull shows the recording positions 1 and 2, from which the traces marked at the left with the same numbers were obtained. The position of the common reference electrode (R) on the nasal bones and the application point of the CSD-eliciting stimulus (KCl) are also shown. CSD was elicited in the frontal cortex by chemical stimulation (a 1- to 2-mm diameter cotton ball soaked with 2% KCl) applied for 1 min on the intact dura mater, as indicated by the horizontal bars. The latencies are shorter in the L_15_ groups compared with the corresponding L_9_ groups. In the groups treated with pilocarpine, the latencies are increased when compared with the respective naïve and vehicle controls. **(B)** CSD propagation velocity. Data are mean ± standard deviation for 8–9 rats per group. ^∗^*p* < 0.001 compared with the corresponding L_9_ condition. #*p* < 0.001 compared with controls groups in the same lactation condition (ANOVA plus Holm-Sidak test).

In all groups, topical application of 2% KCl at one point of the frontal cortical surface for 1 min elicited, as a rule, a single CSD wave that propagated without interruption and was recorded at the two parietal recording points (Figure 4A; see the recording points in the skull diagram). During the 4-h recording session, the slow DC potential change, which consistently appeared after KCl stimulation, confirmed the presence of CSD. In the ECoG recordings, no signs of hyperexcitability (i.e., spikes, sharp waves, paroxysmal depolarization shifts etc.) were observed.

In the L_9_ animals, CSD velocities (mean ± SD in mm/min) in the naïve, vehicle and pilocarpine groups were, respectively, 3.69 ± 0.13, 3.70 ± 0.10, and 3.11 ± 0.15. In L_15_ animals, the CSD velocities for the naïve, vehicle and pilocarpine groups were, respectively, 4.20 ± 0.17, 4.18 ± 0.18, and 2.93 ± 0.23. ANOVA indicated a main effect of the lactation condition [*F*(1,46) = 33.575; *p* < 0.001], and *post hoc* (Holm–Sidak) test comparisons showed that the velocities were higher in the L_15_ groups compared to the L_9_ groups. ANOVA also detected a main effect of treatment [*F*(2,46) = 173.067; *p* < 0.001], and *post hoc* testing revealed that pilocarpine treatment significantly lowered the CSD propagation velocity compared with the corresponding naïve and vehicle controls. The pilocarpine decelerating effect on CSD propagation was more intense in L_15_ than L_9_ rats [*F*(2,46) = 22.658; *p* < 0.001], indicating an interaction between the treatment with pilocarpine and the lactation condition. Data on CSD velocity are shown in Figure 4B.

Table 1 shows data on amplitude and duration of the negative slow potential change, which is the hallmark of CSD. ANOVA indicated a main effect of the lactation condition on the CSD amplitude [*F*(1,42) = 8.165; *p* = 0.007], and a *post hoc* (Holm–Sidak) test comparison showed that the amplitudes were higher in the naïve and vehicle L_15_ groups compared to the corresponding L_9_ groups. The factor treatment also affected the amplitude [*F*(2,42) = 9.490; *p* < 0.001], and a *post hoc* test showed that the amplitude was lower in the L_15_, but not in the L_9_ pilocarpine-treated group, compared with the corresponding naïve and vehicle controls. ANOVA also confirmed an interaction between both factors [*F*(2,42) = 8.999; *p* < 0.001].

**Table 1:** The unfavorable lactation condition increases amplitude and diminishes duration of the negative slow potential change of CSD, whereas chronic application of a sub-convulsing dose of pilocarpine exerts the opposite effect.

Group	Amplitude (mV)	Duration (s)
**L_9_**		
Naïve	8.4 ± 1.8 (9)	69.6 ± 2.3 (9)
Vehicle	8.5 ± 1.1 (9)	70.1 ± 2.6 (9)
Pilocarpine	8.4 ± 1.1 (9)	69.7 ± 1.3 (9)
**L_15_**		
Naïve	11.1 ± 2.5 (6)#	64.4 ± 0.9 (8)#
Vehicle	11.0 ± 1.5 (7)#	64.8 ± 1.1 (9)#
Pilocarpine	7.0 ± 1.2 (8)*	67.2 ± 1.2 (9)*#


*Male rats were previously suckled in litters with 9 and 15 pups (respectively, L_9_ and L_15_ condition). Naïve, no treatment; Vehicle, scopolamine methyl nitrate 1 mg/kg/day dissolved in 0.9% saline + 0.9% saline; Pilocarpine, scopolamine methyl nitrate 1 mg/kg/day + 45 mg/kg/day of pilocarpine; both dissolved in 0.9% saline. Data are expressed as the mean ± standard deviation, with the number of animals in parentheses. ^∗^*p* < 0.001 compared with control groups in the same suckling condition. #*p* < 0.001 compared with the corresponding group in the L_9_ condition (ANOVA plus Holm-Sidak test).*

Analysis of CSD duration indicated a main effect of the lactation condition [*F*(1,47) = 84.197; *p* < 0.001] and treatment [*F*(2,47) = 3.262; *p* = 0.047] as well as an interaction between these two factors [*F*(2,47) = 3.674; *p* = 0.033]. The Holm-Sidak test indicated a shorter duration in the L_15_ groups compared with the corresponding L_9_ groups and a longer duration in the L_15_ pilocarpine-treated animals compared with the corresponding naïve and vehicle controls.

## Discussion

The present data demonstrate that treatment for 21 days with a sub-convulsing dose of pilocarpine reduces glycemia, promotes anxiety-like behavior and decelerates CSD in rats suckled on two litter sizes. In some measurements (see “Results”), the data also suggest an interaction between lactation condition and pilocarpine treatment, indicating that the pilocarpine effect is more evident in the nutritional deficiency condition (L_15_). Data on weight gain and open field behavior using a relatively low, sub-convulsing dose of pilocarpine constitute novel evidence of the pilocarpine action in rats, whereas data on CSD confirm a previous study (Guedes and Vasconcelos, 2008). The stress of the treatment procedure cannot be the cause of the reported alteration because the groups that received intraperitoneal injection of vehicle (saline and scopolamine) presented values similar to the naïve control. The results emphasize the effectiveness of pilocarpine in modifying behavioral and electrophysiological functioning of the brain.

Our findings on body weight difference between the L_9_ and L_15_ lactation condition confirm previous evidence on the efficacy of increasing litter size in producing nutritional deficiency (Francisco and Guedes, 2015; Lima et al., 2017). Increasing the litter size during the suckling period (L_15_ condition) effectively impairs the pups’ nutritional status, as judged by their reduced body weights (Rocha-de-Melo et al., 2006). This is in line with evidence from others indicating that body weight diminution reflects weight reduction in the brain and other important organs, which is usually accompanied by alterations in the organs’ function (Morgane et al., 1978, 1993). Furthermore, the reduction in blood glucose levels in the L_15_ groups, compared with the corresponding L_9_ groups, supports this conclusion.

Regarding the action of pilocarpine, previous work reported impairment in the growth and development as well as high blood glucose levels in pups of pilocarpine-treated epileptic dams (Cossa et al., 2016). In the present work, pilocarpine treatment of the pups did reduce the animals’ body weight (Figure 1) and their blood glucose levels (Figure 2). While the data on body weight are coherent, differences in the blood glucose levels could be attributed to distinct pilocarpine treatment paradigms: administration of convulsing doses to the mothers (Cossa et al., 2016) vs. treatment of the pups with sub-convulsing doses (present work).

The present open field findings (Figure 3) are coherent with our previously published data (Accioly and Guedes, 2017; Lima et al., 2017). In contrast, some studies reported lower anxiety-like behavior in animals suckled in large litters in comparison with the control group (Bulfin et al., 2011; Clarke et al., 2013). This discrepancy could be due to methodological differences such as the number of pups per litter (12 and 20 pups vs. 9 and 15 pups in our work), type of behavioral test (elevated plus maze vs. open field in our work) and age of testing (PND25 or PND77, vs. PND58 in our work). In addition, those authors subjected the animals to other experimental procedures such as anesthesia with isoflurane and imaging (dual energy X-ray absorptiometry system) for whole body composition; this could likely contribute to the differences discussed here.

In the open field test, the treatment with a sub-convulsing dose of pilocarpine was associated with a more anxious behavior than that observed in the control animals (Figure 3). Of relevancy, the cholinergic system is implicated in emotional regulation (Hoeller et al., 2016). Both sub-convulsing (Duarte et al., 2010, 2013; Hoeller et al., 2016) and convulsing doses of pilocarpine (Leite et al., 2016) can induce short-lasting and long-lasting anxiogenic responses in rats. Taken together, these data allow us to suggest an important behavioral role for the cholinergic system and cholinergic drugs, which would lead to low preference for social novelty, as indicated by longer immobility after pilocarpine administration (Castelhano et al., 2015).

Over the last three decades, our group has characterized the accelerating effect of nutritional deficiency on CSD quite well (Guedes et al., 1987; Rocha-de-Melo et al., 2006; Guedes, 2011; Francisco and Guedes, 2015; Accioly and Guedes, 2017; Lima et al., 2017). The present data (Figure 3) confirm this effect, whose underlying mechanisms are still a subject of investigation. When acting during the critical period of brain development, malnutrition can reduce the number and/or size of brain cells, the amount of myelin, the number of synapses and can alter the functioning of neurotransmitter systems (Morgane et al., 2002; Guedes, 2011). Of note, the reduced brain uptake of glutamate (Feoli et al., 2006) and the increased activity of key enzymes such as glutamic acid decarboxylase (Díaz-Cintra et al., 2007) has also been reported in malnourished animals, and both processes facilitate the propagation of CSD (Peeters et al., 2007; Tottene et al., 2009).

The possible relationship between CSD and brain excitability has been investigated in our laboratory using the acute pilocarpine administration paradigm; both convulsing (Guedes and Cavalheiro, 1997) and sub-convulsing dosing with a single injection of pilocarpine (Vasconcelos et al., 2004; Guedes and Vasconcelos, 2008) decelerated CSD. In the present study, we treated young animals (PND35) over 21 days with a sub-convulsing dose of pilocarpine and confirmed its CSD decelerating action. Recently, Mendes-da-Silva and co-workers treated well-nourished and malnourished younger rats (PND7-28) with the same dose of pilocarpine and found similar CSD outcome (Mendes-da-Silva et al., 2018), suggesting that the time-window for the pilocarpine action on CSD is not narrow. These reports are pioneering in demonstrating the action of chronic pilocarpine on CSD both under favorable and unfavorable conditions of lactation. In comparison to the pilocarpine protocol usually described in the literature to provoke seizure in rodents (300–380 mg/kg), the sub-convulsing dose of pilocarpine used in the present work (45 mg/kg) represents, on average, 12–15% of the convulsing dose. At this low dose, no behavioral signs of epilepsy were detected in our animals. Although sub-convulsing, our pilocarpine dose was effective in counteracting CSD propagation, as evaluated by the alteration in CSD parameters (lower propagation velocity, negative DC amplitude, and longer duration) in the pilocarpine-treated group in comparison with the controls. Pilocarpine displaces the balance between neural excitatory and inhibitory mechanisms toward a hyperexcitability state (Morimoto et al., 2004; L’amoreaux et al., 2010), which makes elicitation and propagation of CSD more difficult (Guedes and Cavalheiro, 1997). The relationship between changes in brain excitability and CSD is still a matter of controversy. On one hand, in humans hyperexcitability has been associated with CSD appearance in migraine (Pietrobon and Moskowitz, 2014; Vinogradova, 2018); on the other hand, animal data demonstrate that CSD does not invade a cortical region in which hyperexcitability has been produced by repetitive electrical stimulation (Koroleva and Bureš, 1979). Regarding the probable biochemical mechanisms underlying the CSD impairment that is associated with pilocarpine treatment, one possibility would be based on the metabolic adaptation that increases brain efficiency to remove extracellular potassium under conditions of hyperexcitability (Heinemann and Lux, 1975; Koroleva and Bureš, 1980). Interestingly, hyperexcitability reduction by benzodiazepines restores the CSD proneness of the pilocarpine-induced resistant brain (Guedes and Cavalheiro, 1997). Nevertheless, additional factors, such as the action of excitatory amino acids and the participation of disinhibition mechanisms (Silva-Gondim et al., 2017), that also modulate brain excitability could contribute for the observed CSD effects of pilocarpine. The glutamatergic system, via *N*-methyl-D-aspartate receptors (NMDARs), importantly participates in developmental and excitability processes in the young brain (Szczurowska and Mareš, 2013) and influences CSD (Marrannes et al., 1988). On the other hand, disinhibition mechanisms, via depressing GABAergic interneurons, could also participate in hyperexcitability generation (McMahon and Kauer, 1997) and in CSD, as recently suggested (Silva-Gondim et al., 2017). Furthermore, GABA release has been found to reduce CSD amplitude (Richter et al., 2014). Further investigation shall confirm whether these two mechanisms are involved in the pilocarpine effects and are mutually exclusive or might act together.

Regarding the pilocarpine/malnutrition interaction, Cabral et al. (2011) reported that malnourished rats require a lower pilocarpine dose to become epileptic in comparison with well-nourished animals, suggesting that malnutrition early in life reduces the threshold for pilocarpine-induced epilepsy. Our CSD findings using a sub-convulsing dose of pilocarpine are consistent with these findings (see Figure 4). Taken together, the data confirm the hypothesis of an interaction between pilocarpine and malnutrition in the rat brain.

## Conclusion

In conclusion, this study documents the metabolic, behavioral and electrophysiological effects of a low dose of pilocarpine and suggests that early malnutrition modulates the pilocarpine effects. The findings support the following three conclusions. First, chronic treatment with sub-convulsing doses of pilocarpine produced anxiety-like behavior and reduced the propagation velocity of CSD. Second, increasing litter size caused nutritional deficiency, reduced fasting blood glucose and accelerated CSD, confirming previous studies. Third, unfavorable lactation conditions (L_15_ condition) differentially modulated the pilocarpine effects on blood glucose levels and CSD. The present data might advance understanding the relationship between pilocarpine action on neuronal excitability, anxiety-like behavior and nutritional deficiency.

## Author Contributions

EF performed the experiments, analyzed the data and participated in conceiving the study, and writing and reviewing the manuscript. RG conceived the study, provided the funds, analyzed the data, and wrote the manuscript.

## Conflict of Interest Statement

The authors declare that the research was conducted in the absence of any commercial or financial relationships that could be construed as a potential conflict of interest.
